# Antibiotic resistance, biofilm forming ability, and clonal profiling of clinical isolates of *Staphylococcus aureus* from southern and northeastern India

**DOI:** 10.2478/abm-2022-0023

**Published:** 2022-08-31

**Authors:** Archana Loganathan, Ramesh Nachimuthu

**Affiliations:** Department of Bio-Medical Sciences, School of Bio Sciences and Technology, Vellore Institute of Technology, Vellore, Tamil Nadu 632014, India

**Keywords:** biofilms, drug resistance, bacterial, enterobacterial repetitive intergenic consensus polymerase chain reaction, methicillin-resistant *Staphylococcus aureus*, Mizoram, Tamil Nadu

## Abstract

**Background:**

*Staphylococcus aureus* is a pathogen endemic in India and sometimes deadly for patients in intensive care units.

**Objectives:**

To determine the antibiotic-resistance pattern, biofilm forming ability, and clonal type of *S. aureus* from isolates collected in Tamil Nadu (south) and the Mizoram (northeast) regions of India.

**Methods:**

We collected *S. aureus* isolates from diagnostic laboratories in Tamil Nadu and Mizoram. An antibiotic susceptibility test was performed according to Clinical Laboratory and Standards Institute methods. Antibiotic-resistant determinants such as *mecA*, *mecC*, *blaZ*, *vanA*, *vanB*, and *vanC* were confirmed by polymerase chain reaction (PCR). All isolates were further studied for biofilm forming ability. Enterobacterial repetitive intergenic consensus (ERIC)-PCR was used for clonal analysis.

**Results:**

A study of 206 clinical isolates showed 52.9% prevalence of methicillin-resistant *S. aureus* in Tamil Nadu and 49.4% in Mizoram. Minimum inhibitory concentration tests showed a high prevalence of 67% oxacillin resistance in isolates from Tamil Nadu and 49% in isolates from Mizoram. PCR showed 53% *mecA* in Tamil Nadu and 49% *mecA* in Mizoram. Vancomycin-intermediate resistance *S. aureus* (VISA) prevalence was lower in isolates from Tamil Nadu (4%) and Mizoram (5%). All methicillin-resistant *S. aureus* (MRSA) isolates formed biofilms. Clonal analysis revealed a genetic relatedness between the isolates.

**Conclusions:**

The prevalence of MRSA is high in the regions studied, with most of the clinical isolates being multidrug resistant. Adopting appropriate community-based preventive measures and establishing antimicrobial stewardship is highly recommended to minimize the dissemination in antibiotic resistance.

*Staphylococcus aureus* is one of the ESKAPE pathogens (*Enterococcus faecium*, *Staphylococcus aureus*, *Klebsiella pneumoniae*, *Acinetobacter baumannii*, *Pseudomonas aeruginosa*, and *Enterobacter* species) [1]. These bacteria are known to cause severe hospital-acquired and secondary infections. The advent and spread of antibiotic-resistant *S. aureus*, especially methicillin-resistant *S. aureus* (MRSA) and vancomycin-resistant *S. aureus* (VRSA), pose a global challenge in the treatment of *S. aureus* infections. Newer antibiotics, such as linezolid, tigecycline, and daptomycin, are currently being used to treat MRSA infections [2, 3]. Recently, various nonantibacterial treatments for *S. aureus* infections have been developed, most notably phage therapy for treating VRSA and MRSA [4].

Globally, antibiotic-resistance patterns and the spread of epidemic clones are continuously monitored by the 2 bodies, the U.S. Centers for Disease Control (CDC) and the World Health Organization (WHO). In India, antimicrobial surveillance is monitored by the Indian Network for Surveillance of Antimicrobial Resistance (INSAR) and Antimicrobial Resistance Research and Surveillance Network (ARRSN), which are actively involved in collecting and analyzing the results from various regional centers located across India. The 2020 annual report by the ARRSN has shown that resistance trends in *S. aureus* have increased to most antibiotics over the past years. The report also claims that isolation rates or incidence of *S. aureus* have steadily declined from 2016 to 2020 [5].

India is a geographically wide and densely populated country divided into various regions and states. The distribution of antibiotic-resistant *S. aureus* in India is known to vary by location and time. The overall prevalence of *S. aureus* varies by year, with the highest being 40%–50% observed in 2008 and the lowest prevalence of 33% reported in 2016. Recently, from 2018 to 2020, the incidence of MRSA is known to have reduced despite high resistance [6, 7]. The current resistance trend of *S. aureus* in India is due primarily to community-associated (CA)-MRSA [8]. The INSAR found that CA-MRSA is highly distributed among specimens obtained from throat, genital, nasal, and ear swabs of outpatients, and healthcare-associated (HA)-MRSA was more prominent in skin and soft tissue infections, the bloodstream, catheter tips, fluids from sterile body sites, and lower respiratory tract infections of inpatients [6].

The prevalence of the gene *mecA* in clinical samples among Indian populations is frequently documented; whereas, to our knowledge, there are no reports to date on the prevalence of the *mecC* in clinical samples from India. Similarly, reports of VRSA are scant. From India, only 2 isolates containing *vanA* have been reported up to 2015 [9]. In addition to the presence of resistance factors, biofilm forming infections may be critical and challenging for antibiotic treatment. The ability of *S. aureus* to form biofilms is well-documented [10]. Biofilm forming infections are considered life-threatening because of their complicated architecture that prevents drug penetration. According to a report from the U.S. National Institutes of Health, antibiotic failure may be the cause of death in more than 60% of biofilm forming infections. Therefore, studying the nature of biofilms and their association with antibiotic resistance is critical for addressing biofilm forming infections [11].

The present study aimed to determine the prevalence of MRSA and vancomycin-intermediate *S. aureus* (VISA) in Mizoram and Tamil Nadu. The objectives of the present investigation were to (1) to assess antibiotic susceptibility patterns of *S. aureus* to determine the incidence of MRSA in the regions studied; (2) to investigate the nature of biofilms formed by resistant and sensitive isolates of *S. aureus*; and (3) to assess the genetic relatedness of the bacteria to understand the clonal dissemination of *S. aureus* in Tamil Nadu and Mizoram.

## Methods

### Study design

A total of 206 nonduplicate *S. aureus* isolates was collected during the study period from 2018 to 2019. We conducted this cross-sectional study with the isolates collected from Hi-Tech diagnostic centers in Chennai and Tiruchirappalli, in Tamil Nadu (southern region), and a clinical diagnostic laboratory in Aizawl, in Mizoram (northeastern region). We choose the largest, most densely populated cities in the 2 states and collected isolates at various times throughout 2018 to 2019. The diagnostic centers were selected based on the number and diversity of samples received and ensured that a pan regional representation of isolates was attained. The Institutional Ethical Committee (IEC) of Vellore Institute of Technology, Vellore approved the study protocol (VIT/IECH/004/Jan28.2017). The present study involved laboratory-based analysis of anonymized isolates only. This reporting of the obtained microbiological data is based on the MICRO (Microbiology Investigation Criteria for Reporting Objectively) and STROBE-AMS (Strengthening the Reporting of Observational Studies in Epidemiology-antimicrobial stewardship) statement reporting guideline checklists [12, 13].

### Isolate collection and processing

All *S. aureus* isolates were identified using a VITEK 2 system (bioMérieux) and were transported from the diagnostic centers to the Antibiotic Resistance and Phage Therapy Lab, Vellore Institute of Technology in leak proof, screw capped, cryovial tubes and processed using standard microbiological techniques. Upon receiving the isolates at the Vellore Institute of Technology laboratory facility, we streaked all the isolates onto brain–heart infusion agar. Each isolate was assigned a unique identifier number (SA-1 to SA-206, where SA indicates *Staphylococcus aureus*) and stored in 20% glycerol at low temperature for further analysis. All reported laboratory studies of antibiotic profiling by phenotypic and genotypic analysis were conducted by the Antibiotic Resistance and Phage Therapy Lab, Vellore Institute of Technology. According to the proposed study, MRSA is defined as the presence of *mecA* validated by polymerase chain reaction (PCR).

### Sample source of the isolates

Of 206 *S. aureus* isolates, 119 isolates were collected from Tamil Nadu, and 87 isolates were collected from Mizoram. These isolates were obtained from clinical sources such as pus, sputum, wound sepsis, urine, catheter tip, blood, synovial fluid, throat swab, wound swab, bronchial fluid, umbilical swab, dysuria, and stool. All the isolates were identified using the VITEK 2 GP ID card system (bioMérieux).

### Antibiotic susceptibility testing—disk-diffusion method

Antibiotic susceptibility was tested using a disk-diffusion method. Briefly, the bacterial culture was calibrated to a turbidity of McFarland Standard No. 0.5 (about 1 × 10^8^ colony-forming units [CFU]/mL) and swabbed onto Mueller–Hinton agar (MHA) (HiMedia Laboratories). The plates were allowed to dry for 3 min, and an antibiotic disk was placed on the swabbed surface of the agar. The following antibiotics were tested: oxacillin(30μg), penicillin (10 U), cefoxitin (30 μg), ciprofloxacin (5 μg), levofloxacin (5 μg), ofloxacin (5 μg), tetracycline (30 μg), erythromycin (15 μg), clindamycin (2 μg), linezolid (30 μg), and gentamicin (10 μg) (HiMedia Laboratories).

The methicillin sensitive *S. aureus* subsp. *aureus* Rosenbach (Seattle 1945) from the American Type Culture Collection (ATCC) No. 25923 was used as a quality control strain. Experiments were conducted and interpreted following Clinical and Laboratory Standards Institute (CLSI) guidelines [14]. The antibiotic resistance of each isolate from the disk-diffusion test was recorded independently and multiple-antibiotic resistance (MAR) indices were calculated. The MAR index is defined as “*a*/*b*, where *a* represents the number of antibiotics to which the isolate was resistant, and *b* represents the number of antibiotics to which the isolate was exposed” [15].

### Minimum inhibitory concentration

The minimum inhibitory concentration (MIC) of antibiotics was determined following the CLSI M07 guidelines [14]. Briefly, the MIC of the isolates was determined using a microbroth dilution method in MHA No. 2 cation-controlled broth. The antibiotics oxacillin, ciprofloxacin, and vancomycin were used at concentrations ranging from 128 μg/mL to 0.125 μg/mL. The findings were interpreted following the CLSI guidelines [14]. *S. aureus* ATCC 25923 was used as the standard laboratory testing quality control strain.

The MIC_50_ and MIC_90_ were calculated based on the method described by Schwarz et al. [16] to determine the lowest concentrations of an antibiotic capable of inhibiting 50% and 90% of the bacterial isolate.

### Genomic analysis

Molecular analysis by conventional PCR was used to screen for resistance genes using the following antibiotic-resistant determinants: penicillin-binding protein genes (*mecA* and *mecC*) [17, 18], vancomycin-resistance genes (*vanA*, *vanB*, *vanC*) [19], and β-lactamase gene (*blaZ*) [20]. The primer pairs used in this analysis were synthesized according to sequences published in the literature. The list of primers used in the study is tabulated (**Table 1**). The bacterial DNA was isolated using a phenol–chloroform method, and the extracted DNA was used in the PCR reaction as template DNA. The amplified PCR product was analyzed by 1% agarose gel electrophoresis.

**Table 1:** PCR primers used in the present study

**No.**	**Primer name**	**Primer**	**Reference**
1	*mecA* – F	5′-TCACCAGGTTCAAC[Y] CAAAA-3′	[17]
2	*mecA* – R	5′-CCTGAATC[W] GCTAATAATATTTC-3′	
3	*mecC* – F	5′-GGGTTCAGCCAGATTCATTTGT-3′	[18]
4	*mecC* – R	5′-GTACTGTTGCTTCGTTCAATGG-3′	
5	*vanAB* – F	5′-GTAGGCTGCGATATTCAAAGC-3′	[19]
6	*vanA* – R	5′-CGATTCAATTGCGTAGTCCAA-3′	
7	*vanB* – F	5′-GCCGACAATCAAATCATCCTC-3′	
8	*vanC1* – F	5′-TGGTATTGGTATCAAGGAAACC-3′	
9	*vanC1* – R	5′-AGATTGGAGCGCTGTTTTGTC-3′	
10	*vanC23* – F	5′-CAGCAGCCATTGGCGTACAA-3′	
11	*vanC23* – R	5′-CAAGCAGTTTTTGTAGTAGTTC-3′	
12	*blaZ* – F	5′-TTCAACACCTGCTGCTTTC-3′	[20]
13	*blaZ* – R	5′-CACTCTTGGCGGTTTCAC-3′	

F, forward; PCR, polymerase chain reaction; R, reverse.

### Clonal analysis by enterobacterial repetitive intergenic consensus PCR

Enterobacterial repetitive intergenic consensus (ERIC-PCR was performed using ERIC-1 (5′-ATGTAAGCTCCTGGGGATTCAC-3′) and ERIC-2 (5′-AAGTAAGTGACTGGGGTGAGCG-3′) primers [21]. The amplification conditions were: 95 °C for 1 min, followed by 30 cycles of DNA denaturation at 94 °C for 30 s, annealing at 52 °C for 90 s, extension at 68 °C for 6 min, and final extension at 65 °C for 8 min. The amplified products were separated by 1.5% agarose gel electrophoresis using a 1 kb molecular weight marker ladder to identify multiple band patterns.

### Biofilm analysis—microtiter plate assay

Biofilm formation in vitro was measured by absorption spectroscopy of crystal violet-stained biomass in a 96-well polystyrene microtiter plate assay. Biofilm formation was studied by mimicking the internal body system, in which the polystyrene surface of a 96-well plate was primed with media containing human plasma using a protocol described by Cardile et al. [22]. Briefly, pooled human plasma was diluted to 10% with 50 mM sodium bicarbonate (as a buffering agent). Each well in a microtiter plate was incubated overnight at 4 °C with 100 μL of the 10% plasma. Following the cold incubation, the diluted plasma was removed, and the wells were refilled with 100 μL freshly prepared tryptic soy broth supplemented with 1% glucose. Overnight culture of *S. aureus* was diluted (1:10), and 100 μL of the culture was added to the wells. Biomass was allowed to accumulate at 37 °C for 24 h. After incubation, the wells were gently washed 3× with sterile phosphate-buffered saline (7 mM Na_2_HPO_4_, 3 mM NaH_2_PO_4_, and 130 mM NaCl at pH 7.4) to remove planktonic bacteria and the plates air-dried. Bacteria adherent to the wells were stained for 5 min with 125 μL of a 0.1% solution of crystal violet (Gentian Violet, CAS No. 548-62-9). Finally, 125 μL of 30% acetic acid was added to solubilize the dye, and the optical density (OD) of the well contents determined using a microplate reading spectrophotometer at 570 nm. Acetic acid (30%) served as a blank control, and the broth served as an experimental control. Biofilm formed was categorized as follows: OD test isolate (OD_t_) < OD control blank (OD_c_) was considered no biofilm formation, OD_c_ < OD_t_ < 2 × OD_c_ was considered a weak biofilm producer, 2 × OD_c_ < OD_t_ < 4 × OD_c_ was considered a moderate biofilm producer, and 4 × OD_c_ < OD_t_ was considered a strong biofilm producer.

### Data analysis

All statistical analyses were performed using GraphPad Prism (version 9). MAR indices were determined following the procedure described by Krumperman [15] and Jaja et al. [23]. Significant differences between categorical variables (susceptibility to antibiotic) were determined using a one-way analysis of variance (ANOVA) or a Brown–Forsythe test. The association between the MAR index and antibiotic resistance of isolates from the regions studied was assessed using a χ^2^ test. *P* < 0.05 was considered significant.

DNA fingerprints were analyzed using BioNumerics (version 8.0, Applied Maths, bioMérieux). Gel images were captured in grayscale TIFF format and imported into the Bio-Numerics database to develop a dendrogram. After alignment and normalization, the similarity index was computed and visualized using cluster analysis. For clonal analysis, the Dice coefficients and unweighted pair group average (UPGMA) were used to measure similarity index, with an index of ≥90% being considered the same ERIC type. Banding patterns were defined as present (score = 1) or absent (score = 0).

## Results

### Antibiotic-resistance profiling of *Staphylococcus aureus* in Tamil Nadu

**Figure 1:**
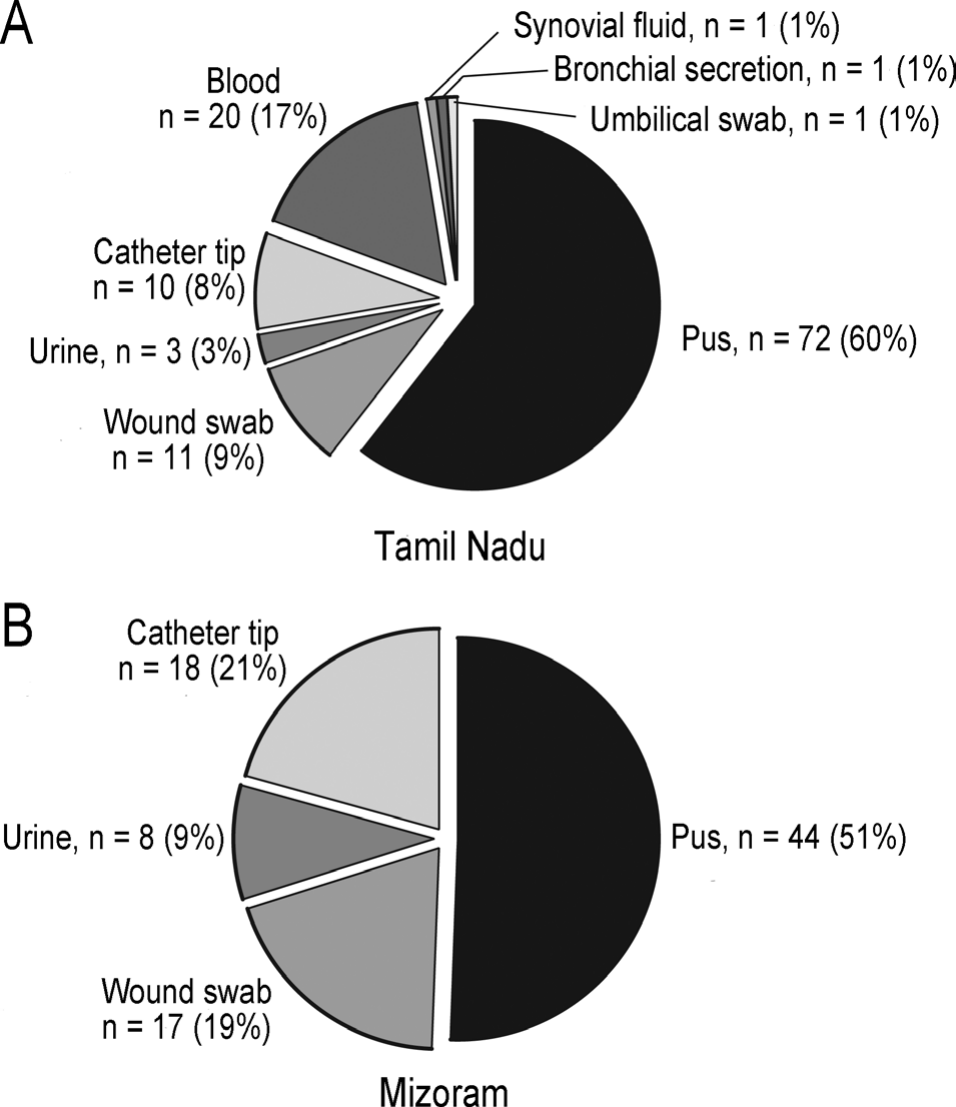
Sample source distribution of isolates from (A) Tamil Nadu and (B) Mizoram.

We collected 119 isolates (SA-1 to SA-119) (57.8%) from clinical diagnostic laboratories in Chennai (n = 114, 96%) and Tiruchirappalli (n = 5, 4%), in Tamil Nadu. The sample sources from which *S. aureus* was isolated included pus, n = 72 (60%), blood, n = 20 (17%), wound swab, n = 11 (9%), catheter tip, n = 10 (8%), urine, n = 3 (3%), and synovial fluid, bronchial secretion, and umbilical swab, n = 1 (1%) each **(Figure 1A)**.

Disk-diffusion tests showed isolates were resistant or susceptible to various antibiotics (**Table 2**). Of the antibiotics tested, isolates showed the highest resistance to penicillin, followed by resistance to erythromycin, and ciprofloxacin, and were highly susceptible to linezolid. MAR indices for the isolates from Tamil Nadu ranged from 0.18 to 0.91, with 118 isolates showing a score >0.25. All 119 isolates were resistant to at least 1 antibiotic under study. The association between the MAR index and antibiotic resistance of isolates from Tamil Nadu was significant (*P* = 0.001). There were 117 (98%) multidrug resistant (MDR) isolates. As observed from the MIC, 80 (67%) isolates showed resistance to oxacillin, 101 (85%) isolates were resistant to ciprofloxacin antibiotic, and none showed vancomycin resistance. However, 5 (4%) isolates were vancomycin-intermediate resistant *S. aureus* (VISA). The MIC_50_ and MIC_90_, respectively, were 1 μg/mL and 0.5 μg/mL for oxacillin, 16 μg/mL and 16 μg/mL for ciprofloxacin, and 2 μg/mL and 2 μg/mL for vancomycin.

**Table 2:** Antibiotic resistance by disk diffusion of isolates from Tamil Nadu and Mizoram

**Antibiotic**	**Antibiotic class**	**Antibiotic resistance of isolates from Tamil Nadu** **n (%)**		**Antibiotic resistance of isolates from Mizoram** **n (%)**	

		**R**	**S**	**R**	**S**
Oxacillin	β-lactam	66 (56)	53 (45)	43 (49)	44 (51)
Penicillin	β-lactam	117 (98)	2 (2)	86 (99)	1 (1)
Cefoxitin	β-lactam	91 (76)	28 (24)	70 (81)	17 (20)
Ciprofloxacin	Fluoroquinolone	98 (82)	21 (18)	65 (75)	22 (25)
Levofloxacin	Fluoroquinolone	66 (56)	53 (45)	61 (70)	26 (30)
Ofloxacin	Fluoroquinolone	75 (63)	44 (37)	57 (66)	30 (35)
Tetracycline	Tetracycline	47 (40)	72 (61)	27 (31)	60 (69)
Erythromycin	Macrolide	104 (87)	15 (13)	60 (69)	27 (31)
Clindamycin	Lincosamide	34 (29)	85 (71)	39 (45)	48 (55)
Linezolid	Oxazolidinone	16 (13)	103 (87)	12 (14)	75 (86)
Gentamicin	Aminoglycoside	73 (61)	46 (39)	30 (35)	57 (66)

R, resistant; S, susceptible.

PCR revealed that 63 (53%) isolates carried *mecA*, 111 (93%) carried *blaZ*, and none carried *mecC*. Vancomycin-resistance genes *vanA*, *vanB*, and *vanC* screened for in the VISA isolates were absent.

**Figure 2:**
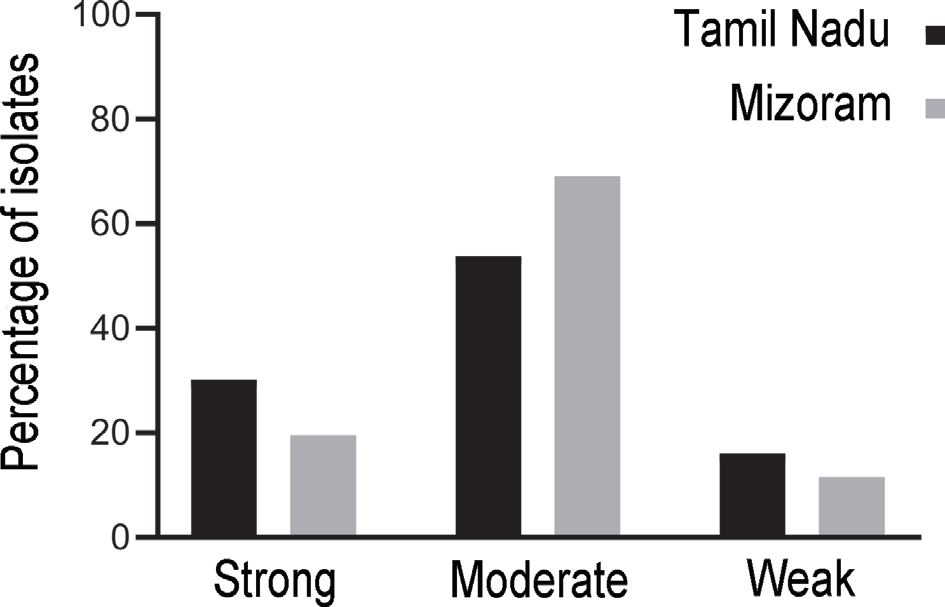
Strength of biofilm formation by the isolates from Tamil Nadu and Mizoram.

Biofilm screening revealed that 36 (30%) isolates formed a strong biofilm, 64 (54%) formed a moderate biofilm, and 19 (16%) formed a weak biofilm (**Figure 2**). The distribution of MRSA among the biofilm-producing isolates was n = 21/36 (58%) among those forming strong biofilm producers, n = 32/64 (50%) among those forming moderate biofilm, and n = 10/19 (53%) among those forming weak biofilm.

We analyzed the 119 isolates from Tamil Nadu for genetic relatedness by multiple banding similarity patterns in ERIC-PCR and found 8 distinct clusters (I–VIII). Clusters I, IV, and VI had the least number of methicillin-resistant strains at 1 each, with I and VI having 1 MRSA and IV having 1 methicillin-susceptible *S. aureus* (MSSA). Clusters II and V each had 3 strains of 1 MRSA and 2 MSSA, and 2 MRSA and 1 MSSA, respectively. Cluster III had 6 strains, 3 of which were MRSA. Cluster VII had the most strains at 99, including 55 MRSA, while Cluster VIII had 5 MSSA strains in total (**Table 3**, and supporting data in figshare, doi: 10.6084/m9.figshare.20036117).

**Table 3:** ERIC-PCR cluster analysis, isolate distribution, and methicillin resistance of isolates from Tamil Nadu and Mizoram

**Tamil Nadu**			**Mizoram**		
	
**Cluster group**	**No. of isolates within the cluster**	**No. of MRSA**	**Cluster group**	**No. of isolates within the cluster**	**No. of MRSA**
Cluster I	1	1	Cluster I	19	10
Cluster II	3	1	Cluster II	15	6
Cluster III	6	3	Cluster III	21	11
Cluster IV	1	0	Cluster IV	23	12
Cluster V	3	2	Cluster V	5	1
Cluster VI	1	1	Cluster VI	1	1
Cluster VII	99	55	Cluster VII	2	1
Cluster VIII	5	0	Cluster VIII	1	1

ERIC-PCR, enterobacterial repetitive intergenic consensus–polymerase chain reaction; MRSA, methicillin-resistant *Staphylococcus aureus*.

### Antibiotic-resistance profiling of *S. aureus* in Mizoram

We collected 87 isolates (SA-120 to SA-206) (42%) from a clinical diagnostic laboratory in Aizawl, Mizoram. The samples from which *S. aureus* was isolated included pus, n = 44 (51%), wound swab, n = 17 (19%), catheter tip, n = 18 (21%), and urine, n = 8 (9%) **(Figure 1B)**.

Of the antibiotics tested in a disk-diffusion test, isolates showed the highest resistance to penicillin, followed by resistance to cefoxitin, and ciprofloxacin, and were highly susceptible to linezolid (**Table 2**). MAR indices for the isolates from Mizoram ranged from 0.18 to 1.00, with 85 isolates showing a score >0.25. The association between the MAR index and antibiotic resistance of isolates from Mizoram was significant (*P* < 0.003). We found MDR in 82/87 (94%) of the isolates from Mizoram. As observed from the MIC tests, 43 (49%) isolates showed resistance to oxacillin, 65 (75%) isolates were resistant to ciprofloxacin, and none showed resistance to vancomycin. However, 4 (5%) of the isolates were VISA. The MIC_50_ and MIC_90_ were, respectively, 0.5 μg/mL and 0.125 μg/mL for oxacillin, 0.125 μg/mL and 0.125 μg/mL for ciprofloxacin, and 4 μg/mL and 4 μg/mL for vancomycin.

PCR revealed that 43 (50%) isolates carried *mecA*, 70 (81%) carried *bla*Z, and none of the isolates carried *mecC, vanA*, *vanB*, or *vanC*.

Biofilm screening revealed that 17 (20%) isolates formed a strong biofilm, 60 (69%) formed a moderate biofilm, and 10 (12%) formed a weak biofilm (**Figure 2**). Prevalence of MRSA in biofilm-producing isolates was n = 7/17 (41%) in those forming a strong biofilm, n = 30/60 (50%) in those forming a moderate biofilm, and n = 6/10 (60%) in those forming a weak biofilm.

ERIC-PCR analysis of the isolates from Mizoram showed 8 distinct clusters (I–VIII). Clusters II and IV had the least number of MRSA strains at 1 each, cluster III had 2 strains, of which 1 was MRSA, cluster I had 5 strains, of which 1 was MRSA and the other 4 were MSSA, cluster VI had 15 strains, with 6 MRSA, cluster V had 19 strains with 10 MRSA, cluster VII had 21 strains with 11 MRSA, and cluster VIII had the highest number of strains at 23, of which 12 were MRSA (**Table 3**, and supporting data in figshare, doi: 10.6084/m9.figshare.20036117).

## Discussion

The present article reports the antibiotic-resistance profiling of *S. aureus* in 2 Indian states: Tamil Nadu in the south and Mizoram in the northeast. We identified a high prevalence of MRSA and MDR isolates in these regions. These data are essential for public health professionals to determine the prevailing pattern of resistance in *S. aureus*, which helps these professionals frame policies to control the development of antibiotic resistance.

One of the key findings of the present study are the high MAR indices in the isolates studied. High MAR indices (>0.25) suggest that the isolates come from high-risk sources where antibiotics are used frequently, which indicates that antibiotic usage in the studied regions was high. The data for antibiotic consumption from the Intercontinental Medical Statistics Health MIDAS (Market Information Data Analytics System) database shows that India is the world's greatest consumer of antibiotics, with the highest consumption observed during 2010, accounting for 10.7 units per person [24, 25]. Similarly, a recent spatial model study has shown that antibiotic consumption in India has increased from 48% in 2000 to 67% in 2018 [26]. The high MAR indices in most of the isolates indicate the need to establish antibiotic stewardship to reduce the overuse and misuse of antibiotics. We also found a high prevalence of MDR isolates in both regions, which is attributed to the overuse of antibiotics in India.

The proportion of oxacillin resistance from the Tamil Nadu region varied considerably according to method in the phenotypic and genotypic analysis. The MIC method found a larger proportion of oxacillin resistance (67%) than PCR (53%). By contrast, for isolates from Mizoram, we found that every oxacillin-resistant *S. aureus* (ORSA) identified by MIC had *mecA*. This variation observed in the isolates of Tamil Nadu is considered to be associated with a borderline oxacillin resistance *S. aureus* (BORSA) phenotype, which results in increased β-lactamase production. In this context, CLSI guidelines discourage the use of oxacillin disk-diffusion tests as a standard method to identify MRSA due to the difference in the inhibitory zone produced by increased β-lactamase production [27]. Due to such phenotypic variations observed in our study, MRSA was reported based on the presence of *mecA* only, which was validated by PCR. The BORSA phenotype predicted by the present study was not explored as this was out of the scope of the present investigation.

In the present study, the prevalence of MRSA was 52.9% in Tamil Nadu and 49.4% in Mizoram. The prevalence of MRSA reported is similar to that in reports of prevalence between 2014 and 2019, which showed that the prevalence of MRSA in India was high at 53% [28]. By contrast, a systemic review conducted on the prevalence of MRSA in various zones of India showed a lower prevalence in southern India with 34% MRSA [29]. The variation in the MRSA prevalence reported in the present study could be attributed to the regions included in the analysis. In the present report from southern India, we only assessed prevalence in Tamil Nadu, while the systemic review reported the prevalence of MRSA from 6 states in southern India: Tamil Nadu, Telangana, Karnataka Andhra Pradesh, Kerala, and Puducherry [29].

MIC by the broth microdilution method revealed that oxacillin resistance was high among the isolates collected from the Tamil Nadu (67%) compared with Mizoram isolates (49%). Overall, MIC revealed high ciprofloxacin resistance, as ciprofloxacin is frequently prescribed in MSSA infection [3]. The prevalence of VISA at 4% in Tamil Nadu and 5% in Mizoram indicate that vancomycin resistance is emerging in these regions. Thus, usage of vancomycin should be restricted to reserve the antibiotic for serious *S. aureus* infections. Tiwari and Sen [30] reported similar prevalence of VISA in the northern part of India. Compared with the prevalence of VISA in Europe and the United States, the prevalence of VISA is higher in Asia [31].

We found that the prevalence of biofilm producers is high in isolates from samples taken in Tamil Nadu. Isolates from Tamil Nadu showed greater prevalence of strong biofilm producers (30%), whereas isolates in Mizoram had comparatively weaker biofilm producers (19%). A study conducted in a tertiary care hospital in the Tripura region in 2015 showed 55% (55/100) of the isolates forming biofilms of various strengths [32].

By contrast, our study of isolates obtained between 2018 and 2019 shows that 100% of the isolates produced biofilms of various strengths. Observing such a high prevalence of biofilm producers could be ascribed to the modified biofilm analysis method used in the present study. However, it remains unclear to what degree the human plasma might affect the strength of the *S. aureus* biofilms. Nevertheless, as consistent with the models of Cardile et al. [22], it can be concluded that adhesive matrix molecules from the plasma enhance biofilm formation by *S. aureus*.

We observed that all MRSA form biofilms of varying strengths, indicating that methicillin resistance and biofilm development have a strong association. Despite this association, there was a significant variation in the strength of the biofilms (strong and moderate biofilms) produced by MRSA, implying that additional factors may influence the strength of the biofilms formed. According to Pozzi et al. [33], virulence mechanisms including biofilm forming capacity and antibiotic resistance are interrelated in MRSA, in which increasing antibiotic resistance directly influences and attenuates potential virulence, thereby resulting in altered biofilm formation. The interrelationship between antibiotic resistance and biofilm formation is known to be governed by homo- and heteroresistance to oxacillin. While the relationship between other antibiotics such as vancomycin and biofilm formation is less explored as resistance due to vancomycin is rarely encountered, the molecular mechanism involved in interrelationship shows that a deletion in the accessory gene regulator (*arg*) system of proteases can enhance biofilm formation by clinical strains of MRSA [34]. However, the precise mechanisms in the interrelationship between antibiotic resistance and biofilm formation are not yet determined [34]. As molecular markers were out of the scope of the present study, their interrelationship with antibiotic resistance was not explored here. Our findings revealed a substantial prevalence of MRSA among strong biofilm producers (58%) in Tamil Nadu. By contrast, isolates from Mizoram revealed a high prevalence of MRSA among weak (60%) and moderate biofilm producers (50%). Overall, the prevalence of biofilm formers and methicillin-resistant biofilm isolates was high in Tamil Nadu. Similarly, for burn patients in Chennai, 75% of isolates were MRSA and biofilm producers [35].

The similarity index of SA-18 (MRSA) and SA-119 (MSSA) of the Tamil Nadu isolates from cluster II shared a typical banding pattern with SA-168 (MRSA) and SA-122 (MSSA) of Mizoram in ERIC-PCR analysis. Similarly, in cluster III, SA-12 (MRSA) and SA-42 (MSSA) shared a related banding pattern with the Mizoram isolates SA-125 (MSSA) and SA-134 (MSSA), respectively. These data show that the rise of antibiotic resistance of isolates from the Mizoram population might be due to medical tourism, shared borders, and human migration, which results in the dissemination of clones by carriers who may unknowingly shed the pathogen during their travel [28].

A strength of this work lies in the detection of MRSA by the presence of *mecA* and the enumeration of the variations observed between the phenotypic and genotypic screenings. Observations of the strengths of the biofilms among the MRSA and clonal dissemination between the 2 regions added background evidence to the study.

We acknowledge several limitations of the present study, notably related to the nonuniformity of isolates from each sample source, many cities not being included in the study as the diagnostic laboratories in those regions refused to share the isolates due to policy restrictions on sharing isolates and secondary data, and the genetic relatedness of isolates being studied by ERIC-PCR (a method not established globally) as it was feasible and cost-effective in our laboratory facility.

## Conclusions

Our molecular typing suggests that the clonal dissemination between Tamil Nadu and Mizoram could play a crucial role in the spread of antibiotic resistance between these regions. Prevalence of *S. aureus* infections in the regions studied are high and emphasize the need for periodic surveillance and monitoring of MRSA outbreaks. In this context, we believe that implementation of a “One Health” approach, in which multiple public health sectors work together to bring about better public health could help control the rise of antibiotic resistance.

## References

[1] De Oliveira DMP, Forde BM, Kidd TJ, Harris PNA, Schembri MA, Beatson SA (2020). Antimicrobial resistance in ESKAPE pathogens. Clin Microbiol Rev.

[2] Bassetti M, Di Biagio A, Cenderello G, Del Bono V, Palermo A, Cruciani M, Bassetti D (2001). Linezolid treatment of prosthetic hip infections due to methicillin-resistant *Staphylococcus aureus* (MRSA). J Infect.

[3] Rayner C, Munckhof WJ (2005). Antibiotics currently used in the treatment of infections caused by *Staphylococcus aureus*. Intern Med J.

[4] Manohar P, Tamhankar AJ, Lundborg CS, Ramesh N (2018). Isolation, characterization and *in vivo* efficacy of *Escherichia* phage myPSH1131. PLoS One.

[5] (2020). Annual Report Antimicrobial Resistance Research and Surveillance Network [Internet].

[6] Joshi S, Ray P, Manchanda V, Bajaj J, Chitnis DS, Indian Network for Surveillance of Antimicrobial Resistance (INSAR) group, India (2013). Methicillin resistant *Staphylococcus aureus* (MRSA) in India: prevalence & susceptibility pattern. Indian J Med Res.

[7] Walia K, Madhumathi J, Veeraraghavan B, Chakrabarti A, Kapil A, Ray P (2019). Establishing antimicrobial resistance surveillance & research network in India: journey so far. Indian J Med Res.

[8] D’Souza N, Rodrigues C, Mehta A (2010). Molecular characterization of methicillin-resistant *Staphylococcus aureus* with emergence of epidemic clones of sequence type (ST) 22 and ST 772 in Mumbai, India. J Clin Microbiol.

[9] Veeraraghavan B, Walia K (2019). Antimicrobial susceptibility profile & resistance mechanisms of Global Antimicrobial Resistance Surveillance System (GLASS) priority pathogens from India. Indian J Med Res.

[10] Senobar Tahaei SA, Stajer A, Barrak I, Ostorhazi E, Szabo D, Gajdacs M (2021). Correlation between biofilm-formation and the antibiotic resistant phenotype in *Staphylococcus aureus* isolates: a laboratory-based study in Hungary and a review of the literature. Infect Drug Resist.

[11] Bryers JD (2008). Medical biofilms. Biotechnol Bioeng.

[12] Tacconelli E, Cataldo MA, Paul M, Leibovici L, Kluytmans J, Schröder W (2016). STROBE-AMS: recommendations to optimise reporting of epidemiological studies on antimicrobial resistance and informing improvement in antimicrobial stewardship. BMJ Open.

[13] Turner P, Fox-Lewis A, Shrestha P, Dance DAB, Wangrangsimakul T, Cusack TP (2019). Microbiology investigation criteria for reporting objectively (MICRO): a framework for the reporting and interpretation of clinical microbiology data. BMC Med.

[14] Clinical and Laboratory Standards Institute (CLSI) (2018). Methods for dilution antimicrobial susceptibility tests for bacteria that grow aerobically.

[15] Krumperman PH (1983). Multiple antibiotic resistance indexing of *Escherichia coli* to identify high-risk sources of fecal contamination of foods. Appl Environ Microbiol.

[16] Schwarz S, Silley P, Simjee S, Woodford N, van Duijkeren E, Johnson AP, Gaastra W (2010). Editorial: assessing the antimicrobial susceptibility of bacteria obtained from animals. J Antimicrob Chemother.

[17] García-Álvarez L, Holden MTG, Lindsay H, Webb CR, Brown DFJ, Curran MD (2011). Methicillin-resistant *Staphylococcus aureus* with a novel *mecA* homologue in human and bovine populations in the UK and Denmark: a descriptive study. Lancet Infect Dis.

[18] Khairalla AS, Wasfi R, Ashour HM (2017). Carriage frequency, phenotypic, and genotypic characteristics of methicillin-resistant *Staphylococcus aureus* isolated from dental health-care personnel, patients, and environment. Sci Rep.

[19] Bell JM, Paton JC, Turnidge J (1998). Emergence of vancomycin-resistant enterococci in Australia: phenotypic and genotypic characteristics of isolates. J Clin Microbiol.

[20] Nadarajah J, Lee MJS, Louie L, Jacob L, Simor AE, Louie M, McGavin MJ (2006). Identification of different clonal complexes and diverse amino acid substitutions in penicillin-binding protein 2 (PBP2) associated with borderline oxacillin resistance in Canadian *Staphylococcus aureus* isolates. J Med Microbiol.

[21] Versalovic J, Koeuth T, Lupski JR (1991). Distribution of repetitive DNA sequences in eubacteria and application to fingerpriting of bacterial genomes. Nucleic Acids Res.

[22] Cardile AP, Sanchez CJ, Samberg ME, Romano DR, Hardy SK, Wenke JC (2014). Human plasma enhances the expression of Staphylococcal microbial surface components recognizing adhesive matrix molecules promoting biofilm formation and increases antimicrobial tolerance *in vitro*. BMC Res Notes.

[23] Jaja IF, Jaja CJI, Chigor NV, Anyanwu MU, Maduabuchi EK, Oguttu JW, Green E (2020). Antimicrobial resistance phenotype of *Staphylococcus aureus* and *Escherichia coli* isolates obtained from meat in the formal and informal sectors in South Africa. Biomed Res Int.

[24] Chaudhry D, Tomar P (2017). Antimicrobial resistance: the next BIG pandemic. Int J Community Med Public Health.

[25] Van Boeckel TP, Gandra S, Ashok A, Caudron Q, Grenfell BT, Levin SA, Laxminarayan R (2014). Global antibiotic consumption 2000 to 2010: an analysis of national pharmaceutical sales data. Lancet Infect Dis.

[26] Browne AJ, Chipeta MG, Haines-Woodhouse G, Kumaran EPA, Hamadani BHK, Zaraa S (2021). Global antibiotic consumption and usage in humans, 2000–18: a spatial modelling study. Lancet Planet Health.

[27] Loganathan A, Manohar P, Eniyan K, Jayaraj R, Nachimuthu R (2019). Evaluation of various phenotypic methods with genotypic screening for detection of methicillin-resistant *Staphylococcus aureus*. Asian Biomed (Res Rev News).

[28] Mogasale VV, Saldanha P, Pai V, Rekha PD, Mogasale V (2021). A descriptive analysis of antimicrobial resistance patterns of WHO priority pathogens isolated in children from a tertiary care hospital in India. Sci Rep.

[29] Patil SS, Suresh KP, Shinduja R, Amachawadi RG, Chandrashekar S, Pradeep S (2022). Prevalence of methicillin-resistant *Staphylococcus aureus* in India: a systematic review and meta-analysis. Oman Med J.

[30] Tiwari HK, Sen MR (2006). Emergence of vancomycin resistant *Staphylococcus aureus* (VRSA) from a tertiary care hospital from northern part of India. BMC Infect Dis.

[31] Zhang S, Sun X, Chang W, Dai Y, Ma X (2015). Systematic review and meta-analysis of the epidemiology of vancomycin-intermediate and heterogeneous vancomycin-intermediate *Staphylococcus aureus* isolates. PLoS One.

[32] Bhattacharya S, Bir R, Majumdar T (2015). Evaluation of multidrug resistant *Staphylococcus aureus* and their association with biofilm production in a tertiary care hospital, Tripura, northeast India. J Clin Diagn Res.

[33] Pozzi C, Waters EM, Rudkin JK, Schaeffer CR, Lohan AJ, Tong P (2012). Methicillin resistance alters the biofilm phenotype and attenuates virulence in *Staphylococcus aureus* device-associated infections. PLoS Pathog.

[34] McCarthy H, Rudkin JK, Black NS, Gallagher L, O’Neill E, O’Gara JP (2015). Methicillin resistance and the biofilm phenotype in *Staphylococcus aureus*. Front Cell Infect Microbiol.

[35] Ramakrishnan M, Putli Bai S, Babu M (2016). Study on biofilm formation in burn wound infection in a pediatric hospital in Chennai, India. Ann Burns Fire Disasters.

